# Renal Resistive Index as a Prognostic Indicator in Patients With Hepatic Cirrhosis: An Analytical Cross-Sectional Study

**DOI:** 10.7759/cureus.82700

**Published:** 2025-04-21

**Authors:** Prabhakar K, Anitha A, Sunayana Y Reddy

**Affiliations:** 1 General Medicine, Sri Devaraj Urs Medical College, Kolar, IND; 2 Medicine, Sri Devaraj Urs Medical College, Kolar, IND; 3 General Medicine, Sri Devaraj Academy of Higher Education and Research, Kolar, IND

**Keywords:** chronic liver disease, cirrhosis complications, cirrhosis of liver, doppler ultrasonography, hepatorenal syndrome, intrarenal doppler, liver cirrhosis, non-invasive renal marker, renal dysfunction, renal resistive index

## Abstract

Introduction

Cirrhosis of the liver is a chronic condition marked by scarring and the formation of abnormal nodules, which progressively disrupt normal liver function. In some individuals with cirrhosis, kidney dysfunction may develop without an obvious cause, a condition known as hepatorenal syndrome (HRS). The renal resistive index (RRI), measured via Doppler ultrasound, is a parameter used to assess blood flow resistance in the kidneys. This study aimed to assess the association between the renal resistive index and cirrhosis of the liver and to determine whether intrarenal Doppler can help indicate the presence of hepatorenal syndrome in such cases.

Methodology

An analytical, comparative cross-sectional study was conducted during the study period of August to October 2024 at the Department of Medicine, RL Jalappa Hospital, involving 20 patients diagnosed with cirrhosis and 20 healthy controls. Individuals with conditions such as diabetic nephropathy, renal artery stenosis, urinary tract infections (UTIs), urinary abnormalities, hypotension, bradycardia, and haemoglobinopathies were excluded. All participants underwent clinical evaluation, renal function tests, and intrarenal Doppler ultrasonography. Appendix 1 shows case sheet proforma. Sociodemographic and clinical data were collected using a structured questionnaire. Doppler measurements of peak systolic and end-diastolic velocities were used to calculate the RRI. Scans were performed by a single radiologist after overnight fasting. Data analysis was carried out using IBM Corp. Released 2013. IBM SPSS Statistics for Windows, Version 22.0. Armonk, NY: IBM Corp.

Results

Approximately 18 (45%) of both cirrhosis cases and healthy individuals were aged over 60 years. The majority of participants were male. The mean serum bilirubin level was 3.17 mg/dL in cirrhosis cases and 1.1 mg/dL in healthy individuals. The mean international normalized ratio (INR) was 2.1 in cirrhosis cases and 1.3 in healthy individuals. Ascites was present in 11 (55%) and hepatic encephalopathy in 10 (50%) of the cirrhosis cases. The mean renal resistive index was 0.77 in cirrhosis patients and 0.61 in healthy individuals.

Conclusion

This study highlights the significant role of intrarenal RI measurement as a potential predictor in patients with liver cirrhosis. It may serve as a non-invasive tool for early detection and regular monitoring of patients at risk of developing renal impairment, including hepatorenal syndrome.

## Introduction

Hepatic cirrhosis is a chronic liver condition marked by widespread fibrosis and the transformation of normal hepatic tissue into abnormally structured nodules, a process that can evolve over weeks to years [1]. Hepatorenal syndrome (HRS) refers to renal failure in patients with liver disease, occurring without any identifiable clinical, laboratory, or anatomical cause of kidney dysfunction [2].

HRS represents a type of functional renal failure in individuals with cirrhosis, sharing similar mechanisms with prerenal azotemia. Notably, renal arterial vasoconstriction may persist for extended periods of time, lasting weeks or even months before any notable rise in serum creatinine or blood urea nitrogen levels becomes apparent [3].

The development of HRS is often driven by cirrhosis and portal hypertension, which activate a neurohormonal cascade contributing to renal dysfunction. As cirrhosis progresses, it leads to a decline in cardiac output and systemic vascular resistance, perpetuating a cycle of worsening renal vasoconstriction. This ultimately results in diminished renal perfusion and eventual kidney failure [4].

The renal resistive index (RRI) is a Doppler-based ultrasound measure that reflects intrarenal arterial resistance, calculated using the formula (peak systolic velocity - end-diastolic velocity)/peak systolic velocity. Normal RRI values range from 0.50 to 0.70, with elevated values indicating a poorer prognosis in several renal pathologies [5].

RI has also emerged as a potential indicator of intrarenal hemodynamic changes and may be elevated even in cirrhotic patients without overt azotemia, making it a possible early marker of functional renal impairment in this population [6].

Early identification of renal dysfunction in cirrhosis is clinically significant, as it enables timely intervention and may improve patient outcomes.

Objective

To assess the association between renal resistive index and liver cirrhosis and to evaluate the utility of intrarenal Doppler in identifying hepatorenal syndrome.

## Materials and methods

An analytical, comparative, cross-sectional study was conducted during the study period of August to October 2024 in the Department of Medicine at RL Jalappa Hospital, Sri Devaraj Urs Medical College, Tamaka, Kolar. The study included 40 participants who were categorized into two groups: one consisting of patients diagnosed with hepatic cirrhosis and the other comprising healthy individuals serving as controls.

The sample size was estimated based on the difference in mean RRI between cirrhotic patients and controls, as reported by Vinodh et al. [2], which were 0.63 ± 0.03 and 0.54 ± 0.02, respectively. Using these values and applying a 95% confidence level and 90% power, the calculated sample size was 18 per group, as determined using the formula for comparing two means and the MedCalc sample size software. Accounting for a 10% nonresponse rate, the sample size was adjusted to approximately 20 participants in each group. The formula used was N = 2SD²(Zα/2 + Zβ)² / d², where Zα/2 is the critical value of the normal distribution at α/2 (1.96 for 95% confidence), Zβ is the critical value for β (1.28 for 90% power), SD is the standard deviation from previous data, and "d" is the expected difference between the two means.

The study ultimately enrolled 20 individuals with hepatic cirrhosis and 20 healthy controls. Healthy controls were selected from individuals attending the outpatient department for routine health check-ups, matched for age and sex, and confirmed to have no liver, renal, or systemic illnesses. Participants with conditions such as diabetic nephropathy, renal artery stenosis, urinary tract infections (UTIs), structural urinary tract abnormalities, hypotension, bradycardia, and hemoglobinopathies were excluded.

A structured proforma was used to record each participant’s medical history and physical examination findings. The questionnaire was developed by the research team and focused on sociodemographic data and clinical features relevant to cirrhosis and renal function. It was reviewed by subject experts and pretested on a small group of patients to ensure clarity and content validity. All subjects underwent renal function tests (RFT) via semi-automated clinical chemistry analyzers and renal Doppler ultrasonography. Data collection included demographic details, disease history, duration of cirrhosis, medication use, and symptoms suggestive of kidney involvement. Anthropometric measurements were obtained using standardized equipment, and blood pressure (BP) was measured three times in the supine position, with the mean value recorded after at least 10 minutes of rest.

Additionally, 10 ml of venous blood was drawn for analysis of serum creatinine, glycated haemoglobin (HbA1c), and fasting lipid profile. The estimated glomerular filtration rate (eGFR) was calculated using the chronic kidney disease epidemiology collaboration (CKD-EPI) formula.

To minimize bowel gas interference during imaging, participants fasted for at least eight hours prior to the Doppler scan. Peak systolic velocity (PSV) and end-diastolic velocity (EDV) were measured from the interlobar arteries of both kidneys using M-mode Doppler. Three to five consistent waveforms were collected from each kidney. All sonographic evaluations were conducted by the same experienced radiologist to ensure consistency and eliminate inter-observer variation.

The Doppler ultrasonography was performed using a Philips EPIQ 5 ultrasound system. A curvilinear transducer (3.5-5 MHz) was primarily used in adult patients, for individuals with leaner body habitus, a higher-frequency linear transducer (6-12 MHz) was employed to improve flow detection. A 0° Doppler angle was maintained during spectral waveform acquisition from the interlobar arteries to ensure accurate resistive index measurements.

Prior to commencement, ethical approval was obtained from the institutional ethics committee, and informed consent was secured from all participants.

The collected data were compiled using Microsoft Excel (Redmond, USA) and analyzed using the Statistical Package for IBM Corp. Released 2013. IBM SPSS Statistics for Windows, Version 22.0. Armonk, NY: IBM Corp. Categorical variables were expressed as counts and percentages. Statistical significance between groups was evaluated using the chi-square test. Continuous variables were assessed for normality prior to analysis. As the data were approximately normally distributed, values were expressed as mean ± standard deviation (SD), and comparisons between groups were performed using the independent samples t-test. A p-value less than 0.05 was considered statistically significant.

## Results

Demographic characteristics

The mean age in the hepatic cirrhosis group was 58.4 ± 9.7 years, while in the healthy individuals, it was 50.6 ± 10.53 years. In the hepatic cirrhosis group, two participants (10%) were aged 31-40 years, none were in the 41-50 year age group, nine (45%) were between 51-60 years, and nine (45%) were above 60 years. Similarly, nine (45%) of healthy individuals were also aged above 60 years. In both groups, the majority of participants were male, accounting for 36 out of 40 (90%) in total (Table 1).

**Table 1:** Comparison of age and sex distribution between cirrhotic patients and healthy individuals P-values for age groups were calculated using the chi-square test. P-values for sex distribution were obtained using Fisher’s exact test due to small, expected frequencies; p<0.001: highly significant; p<0.05: significant

Variable	Category	Hepatic Cirrhosis n (%)	Healthy Individuals n (%)	Test used	p-value
Age group	31 to 40 years	2 (10.0)	1 (5.0)	Chi-square test	0.49
	41 to 50 years	0 (0.0)	2 (10.0)		
	51 to 60 years	9 (45.0)	8 (40.0)		
	>60 years	9 (45.0)	9 (45.0)		
Sex	Male	18 (90.0)	18 (90.0)	Fisher’s exact test	1
	Female	2 (10.0)	2 (10.0)		

Clinical features of cirrhosis

Ascites was observed in 11 (55%) of the cirrhosis cases, while none of the healthy individuals showed evidence of ascites (p < 0.001). Hepatic encephalopathy was present in 10 (50%) of the cirrhosis group, whereas none of the healthy subjects were affected (p < 0.001). Additionally, HRS was detected in 14 (70%) of the cirrhosis cases, with no cases observed among the healthy individuals (p < 0.001) (Table 2).

**Table 2:** The distribution of clinical features is compared between cirrhosis patients and healthy individuals Fisher’s exact test applied; p<0.001: highly significant; p<0.05: significant

Variables	Category	Hepatic cirrhosis count (%)	Healthy individuals count (%)	p-value
Ascites	No	9 (45.0)	20 (100)	<0.001
	Yes	11 (55.0)	0	
Hepatic encephalopathy	No	10 (50.0)	20 (100.0)	<0.001
	Yes	10 (50.0)	0	
Hepatorenal syndrome	No	6 (30.0)	20 (100.0)	<0.001
	Yes	14 (70.0)	0	

Biochemical parameters and disease severity scores

The mean serum bilirubin level was significantly higher in cirrhosis cases (3.17 ± 1.6 mg/dL) compared to healthy individuals (1.1 ± 0.17 mg/dL, p < 0.001). The mean international normalized ratio (INR) was 2.1 ± 0.3 in cirrhosis cases versus 1.3 ± 0.15 in healthy subjects (p < 0.001). The RRI was also elevated in the cirrhosis group (0.77 ± 0.11) compared to healthy individuals (0.61 ± 0.15, p < 0.001) (Table 3).

**Table 3:** Laboratory parameters and scoring indices and scores are compared between cirrhosis patients and healthy individuals Independent samples t-test; p < 0.001: highly significant; p < 0.05: significant

Category	Hepatic cirrhosis (Mean ± SD)	Healthy individuals (Mean ± SD)	T-value	p-value
Serum bilirubin	3.17 ± 1.6	1.1 ± 0.17	5.75	<0.001
International normalized ratio	2.1 ± 0.3	1.3 ± 0.15	10.67	<0.001
Renal resistive index	0.77 ± 0.11	0.61 ± 0.15	3.85	<0.001
Prothrombin time	3.29 ± 0.4	0.3 ± 0.13	31.81	<0.001
Serum albumin	3.41 ± 0.4	3.5 ± 0.4	-0.71	0.52
Model for end-stage liver disease score	33.2 ± 4.1	6.45 ± 1.3	27.82	<0.001
Child-Pugh score	7.85 ± 1.04	5.25 ± 0.72	9.19	<0.001

Prothrombin time (PT), Model for End-Stage Liver Disease (MELD) score, and Child-Pugh (CP) score were significantly higher in cirrhosis cases (3.29 ± 0.4, 33.2 ± 4.1, and 7.85 ± 1.04, respectively) than in healthy controls (0.3 ± 0.13, 6.45 ± 1.3, and 5.25 ± 0.72, respectively; all p < 0.001). In contrast, the mean serum albumin levels did not differ significantly between the two groups (3.41 ± 0.4 vs. 3.5 ± 0.4, p = 0.52).

Diagnostic performance of RRI based on ROC analysis 

**Figure 1:**
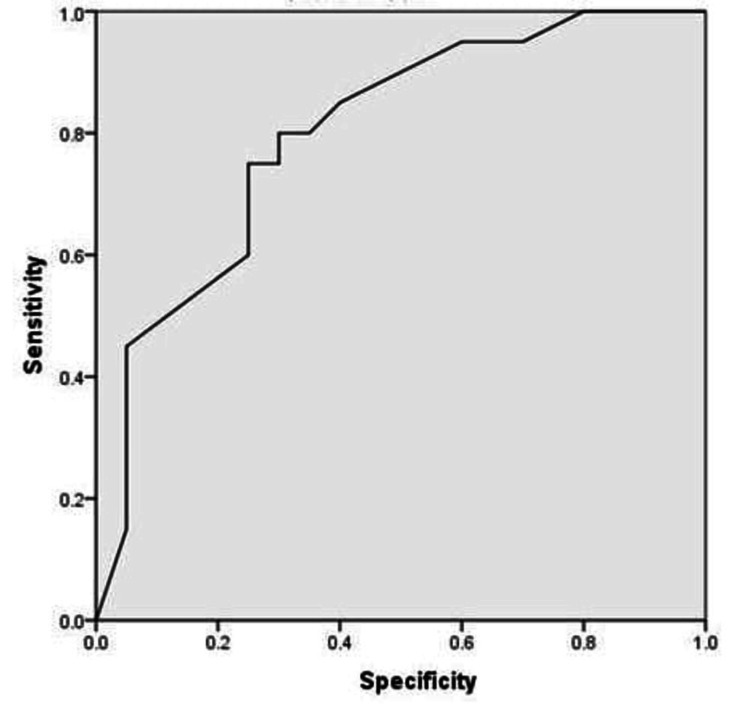
Receiver operating characteristic analysis of Renal Resistive Index Diagonal segments are produced by ties.

Receiver operating characteristic (ROC) analysis was performed to assess the diagnostic utility of the renal resistive index (RRI) in identifying patients with hepatic cirrhosis. The AUC was 0.800 (95% CI: 0.661-0.939; p = 0.001), indicating good discriminatory ability (Figure 1).

At an RRI cut-off of 0.68, the sensitivity and specificity were 80% and 70%, respectively. The positive predictive value (PPV) was 72.7%, the negative predictive value (NPV) was 77.8%, and the overall diagnostic accuracy was 75%.

These values demonstrate that RRI has potential as a non-invasive marker for early renal hemodynamic alterations in cirrhosis (Table 4).

**Table 4:** Cutoff values and predictive accuracy of RRI RRI: Renal Resistive Index

Cut off value of RRI	Sensitivity (%)	Specificity (%)
0.39	100	5
0.45	100	20
0.575	95	40
0.68	80	70
0.715	75	75

## Discussion

This study was undertaken to evaluate the RRI as a sensitive and early prognostic marker of functional renal impairment in patients with hepatic cirrhosis.

Participants aged over 60 years constituted approximately 45.0% of both the cirrhosis and healthy control groups. The majority of participants were male. Among cirrhotic patients, the mean serum bilirubin level was 3.17 mg/dL, compared to 1.1 mg/dL in healthy individuals. The mean INR was 2.1 in cirrhosis cases and 1.3 in controls. Ascites was observed in 55.0% of cirrhotic patients, and hepatic encephalopathy was present in 50.0%. In the study by Vinodh et al. [2], hepatic encephalopathy was also reported among cirrhotic patients with ascites.

Our study demonstrated a significantly higher mean RRI in patients with liver cirrhosis (0.77 ± 0.11) than in healthy controls (0.61 ± 0.15), with a p-value of 0.001. This elevation in RRI suggests early intrarenal hemodynamic changes that may precede elevations in conventional renal markers such as serum creatinine. Several previous studies have reported similar findings.

Goyal et al. [6] reported an RRI of 0.62 in cirrhotic patients versus 0.52 in controls (p < 0.01). Among cirrhotics, those with ascites had an RRI of 0.70, while those without ascites had 0.62. Notably, an RRI above 0.70 was found to be a significant predictor of future HRS (p = 0.006). These findings are consistent with ours, as we also observed elevated RRI in cirrhotic patients with complications such as ascites and encephalopathy.

Mogawer et al. [5] studied 50 cirrhotic patients and found that an RRI measured at the renal artery hilum was strongly associated with HRS. An RRI cut-off value of >0.77 had 100% sensitivity and 66.7% specificity for predicting HRS. Logistic regression revealed that RRI was an independent predictor, with an odds ratio of 35.4 (p = 0.033), supporting our observation that RRI may indicate early renal dysfunction even in the absence of elevated serum creatinine.

Ghoyal et al. [6] evaluated 100 cirrhotic patients and 20 healthy controls. RRI values were 0.52 ± 0.02 in controls, 0.62 ± 0.06 in cirrhotics without ascites, and 0.72 ± 0.02 in those with ascites. In patients with established HRS, RRI averaged 0.74 ± 0.04. Those who developed HRS during follow-up had a baseline RRI of 0.72 ± 0.05, while those who did not had 0.63 ± 0.04 (p < 0.05). These values are closely aligned with our findings in patients showing early renal compromise.

Although Viazzi et al. [4] focused on hypertensive patients, they observed that RRI values above 0.70 were associated with impaired intrarenal blood flow and systemic vascular conditions such as atherosclerosis. They proposed that RRI may serve as a general marker of vascular health, which may explain elevated RRI in cirrhotic patients with normal serum creatinine, as seen in our study.

Chmielewski et al. [3] emphasized the functional nature of HRS, where renal blood flow diminishes despite no structural kidney damage. They advocated for the use of Doppler ultrasonography to detect early vasoconstriction reflected by increased RRI before serum markers like creatinine or estimated glomerular filtration rate (eGFR) become abnormal. This supports our observation that RRI can rise early in the course of renal impairment.

Regmi et al. [1] reported that RRI correlated with liver disease severity. Mean RRI values were 0.78 ± 0.02 in patients with HRS, 0.69 ± 0.03 in cirrhosis without HRS, and 0.60 ± 0.02 in healthy controls (p < 0.001). Significant correlations were also found between RRI and both Model for End-Stage Liver Disease (MELD) and Child-Pugh (CP) scores, which were also observed in our dataset.

Surya et al. [7] studied 200 cirrhotic patients and found higher RRI in those with acute kidney injury (AKI) (0.72 ± 0.06) compared to those without (0.60 ± 0.08, p < 0.001). The highest RRI was observed in patients with acute tubular necrosis (ATN-AKI) at 0.80 ± 0.02, followed by HRS-AKI at 0.73 ± 0.03, and prerenal AKI at 0.63 ± 0.07. They also found positive correlations with MELD-Na and CP scores, as well as increased mortality risk (odds ratio 3.18). These findings are in agreement with our results, supporting RRI as an early marker of renal dysfunction and a prognostic tool in cirrhosis.

Tăluță et al. [8] highlighted limitations in using serum creatinine as a renal marker in cirrhotic patients due to factors like low muscle mass, reduced hepatic creatine synthesis, and bilirubin interference. This aligns with our findings, where many patients had elevated RRI despite normal creatinine levels. Their review also emphasized the role of emerging biomarkers such as neutrophil gelatinase-associated lipocalin (NGAL), kidney injury molecule-1 (KIM-1), interleukin-18 (IL-18), and non-invasive imaging tools such as Doppler ultrasound and shear wave elastography for earlier detection of HRS.

Ozturk et al. [9] similarly discussed the unreliability of serum creatinine in cirrhosis due to malnutrition, muscle wasting, and bilirubin interference. They highlighted that AKI in cirrhosis involves circulatory dysfunction and systemic inflammation, which supports the use of RRI as an early indicator. They also referenced the updated International Club of Ascites (ICA) definition of HRS, which no longer relies on a fixed creatinine threshold, further validating the need for non-invasive, dynamic markers such as RRI.

Sikarwar et al. [10] evaluated RRI across cirrhosis stages and found values of 0.53 in compensated cirrhosis, 0.62 in diuretic-responsive ascites, 0.64 in refractory ascites, and 0.75 in HRS, all significantly higher than controls (0.54, p < 0.05). RRI increased progressively with disease severity, mirroring our findings. They also reported a positive correlation between blood urea and RRI, similar to the associations we observed.

To summarize, our findings are consistent with the literature in showing that RRI increases with worsening liver disease and serves as a sensitive, non-invasive marker for early renal dysfunction. Most importantly, RRI values above 0.70-0.77 appear to be a critical threshold for identifying patients at risk for HRS and related complications.

Limitations

This study has some limitations. First, the sample size was small and derived from a single centre, which may limit the generalizability of the findings. Second, due to its cross-sectional design limits conclusions regarding causality or temporal progression of renal dysfunction. Third, Doppler measurements of RRI are operator-dependent, and we did not assess inter-observer variability. Lastly, the study did not incorporate newer renal biomarkers or subclassify patients based on AKI subtypes, which may have provided additional clinical insights.

Additionally, certain potential confounding variables, such as blood pressure, cardiac output, and liver disease severity scores (e.g., MELD, Child-Pugh), may have influenced RRI values despite our efforts to standardize measurements. These were not adjusted statistically and may have impacted the observed associations. Furthermore, the operator dependency of Doppler measurements, although minimized by a single-radiologist protocol, limits reproducibility in broader settings. Future studies with longitudinal follow-up, multicenter recruitment, and integration of emerging biomarkers such as NGAL and KIM-1 may help validate and expand upon these findings.

## Conclusions

Our study demonstrates that the Renal Resistive Index (RRI) is significantly elevated in patients with cirrhosis, particularly in those with advanced or complicated disease. Compared to healthy individuals, cirrhotic patients exhibit higher RRI values, suggesting its potential as an early predictor of renal dysfunction. Importantly, RRI can identify patients at risk of developing hepatorenal syndrome (HRS), a serious complication of advanced cirrhosis characterized by functional renal failure. The current diagnostic criteria for renal failure in cirrhosis primarily detect cases with markedly reduced glomerular filtration rate (GFR). In contrast, RRI provides an opportunity to detect early renal impairment even before changes in estimated GFR (eGFR) become evident. As a non-invasive, accessible, and reliable tool, RRI holds promise for routine evaluation of renal function in cirrhotic patients. Elevated RRI values may also serve as a warning sign for impending acute kidney injury (AKI), enabling timely interventions and potentially improving outcomes.

## Figures and Tables

**Figure 2:**
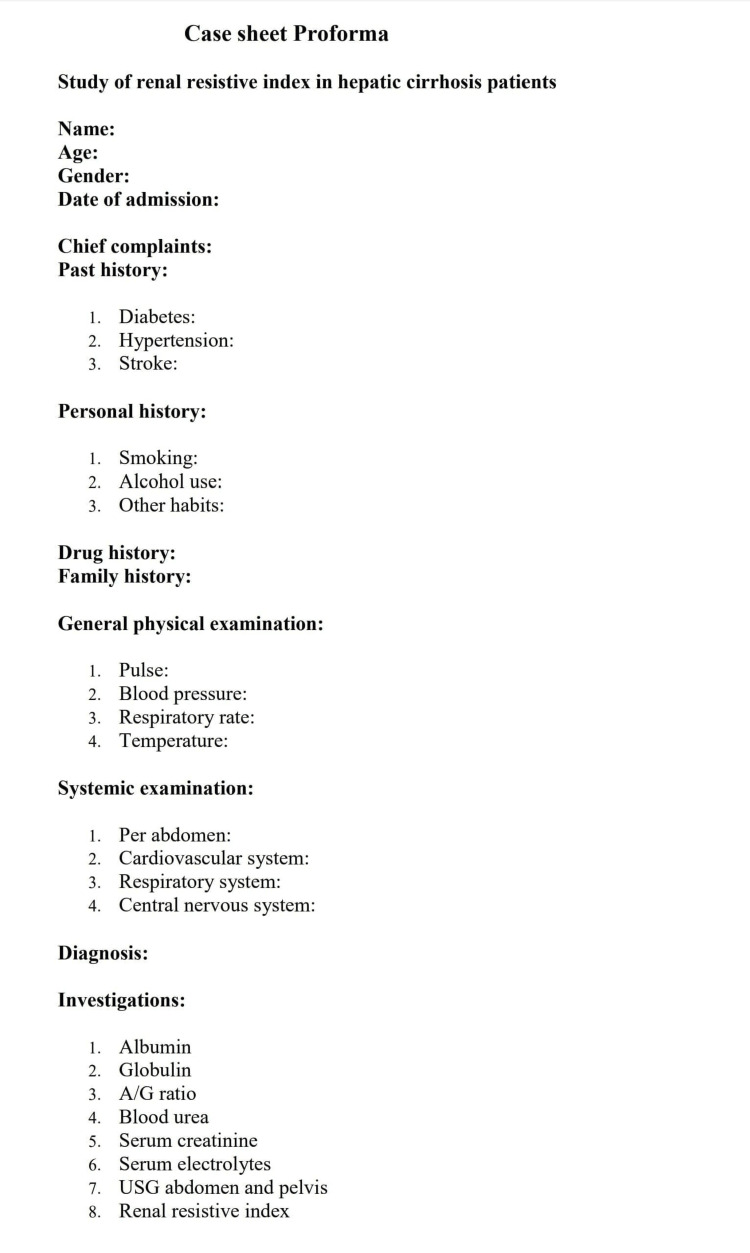
Case sheet proforma

## References

[1] Regmi B, Kunwar L, Khatiwada P (2022). Association of chronic liver disease with resistive index of intra-renal artery. J Clin Images Med Case Rep.

[2] Vinodh V (2018). A study of renal resistive index in hepatic cirrhosis. Int J Contemp Med Surg Radiol.

[3] Chmielewski J, Lewandowski RJ, Maddur H (2018). Hepatorenal syndrome: physiology, diagnosis and management. Semin Intervent Radiol.

[4] Viazzi F, Leoncini G, Derchi LE, Pontremoli R (2014). Ultrasound Doppler renal resistive index: a useful tool for the management of the hypertensive patient. J Hypertens.

[5] Mogawer MS, Nassef SAR, Elhamid SMA (2021). Role of renal duplex ultrasonography in evaluation of hepatorenal syndrome. Egypt Liver J.

[6] Ghosh J (2013). Intrarenal resistance index (RI) as a predictor of early renal impairment in patients with liver cirrhosis. Trop Gastroenterol.

[7] Surya H, Kumar R, Priyadarshi RN (2024). Renal resistive index measurements by ultrasound in patients with liver cirrhosis: Magnitude and associations with renal dysfunction. World J Radiol.

[8] Tăluță C, Ștefănescu H, Crișan D (2024). Seeing and sensing the hepatorenal syndrome (HRS): the growing role of ultrasound-based techniques as non-invasive tools for the diagnosis of HRS. Diagnostics (Basel).

[9] Ozturk NB, Dinc EJ, Swami A, Gurakar A (2023). Acute kidney injury and hepatorenal syndrome in patients with cirrhosis. J Clin Med.

[10] Sikarwar JS, Muchhoria S, Singh R (2014). Study of resistive index in various stages of liver cirrhosis and its significance in calculating the risk for hepatorenal syndrome. J Evol Med Dent Sci.

